# An Efficient Laser Decontamination Process Based on Non-Radioactive Specimens of Nuclear Power Materials

**DOI:** 10.3390/ma16247643

**Published:** 2023-12-14

**Authors:** Yang Hu, Changsheng Liu, Kangte Li, Jian Cheng, Zhiming Zhang, Enhou Han

**Affiliations:** 1 Key Laboratory for Anisotropy and Texture of Materials (Ministry of Education), School of Materials Science and Engineering, Northeastern University, Shenyang 110819, China; 2100516@stu.neu.edu.cn (Y.H.); 2270456@stu.neu.edu.cn (K.L.); 2 Institute of Corrosion Science and Technology, Guangzhou 510530, China; zmzhang@icost.ac.cn (Z.Z.); ehhan@icost.ac.cn (E.H.); 3 Laser Group, School of Mechanical Engineering, Hubei University of Technology, Wuhan 430068, China; chengjian@hbut.edu.cn

**Keywords:** laser decontamination, process without overlap, high efficiency, Alloy 690

## Abstract

Nuclear power components contain radioactivity on their surfaces after long-term service, which can be harmful to personnel and the environment during maintenance, dismantling, and decommissioning. In this experiment, laser decontamination technology is utilized to remove radioactivity from their surfaces. In order to meet the actual needs, a laser decontamination process without spot overlapping has been studied. Under the same equipment conditions, the decontamination efficiency of the non-spot overlapping process is 10 times higher than that of the spot overlapping process. Alloy 690 is used as the test substrate, and non-radioactive specimens are prepared by simulating primary-circuit hydrochemical conditions. The surface morphology, elemental composition, and phase composition of the specimens before and after laser decontamination are investigated with SEM and XRD using the single-pulse experiment and power single-factor experiment methods, and the laser decontamination effect was evaluated. The results show that the decontamination efficiency reached 10.8 m^2^/h under the conditions of a pulse width of 500 ns, a laser repetition frequency of 40 kHz, a scanning speed of 15,000 mm/s, and a line spacing of 0.2 mm, according to which the removal effect was achieved when the laser power was 160 W and the oxygen content on the surface was 6.29%; additionally, there were no oxide phases in the XRD spectra after decontamination. Therefore, the laser cleaning process without spot overlap can provide reference for future practical operations to achieve efficient removal of radioactivity from nuclear power components.

## 1. Introduction

For pipelines and vessels in long-term service in primary circuits, a layer of oxide grows on the surface of their inner walls due to the effects of high temperatures, high pressures, and hydrochemical media [1,2]. This layer of oxide is often loose and porous, providing an opportunity for the invasion of various radionuclides (Cs, Co, Eu and Ce) in the primary loop media, and these components therefore contain high levels of radioactivity. When these components are faced with maintenance, decommissioning, and recycling, we will have to address the issue of removing their radioactivity in order to avoid the hazards these components pose to people and the environment. Accelerator driven system (ADS) is a method for nuclear waste destruction [3]. It uses high-energy protons accelerated by accelerators to undergo spallation reactions with heavy target nuclei (such as lead), and uses neutrons generated to transmute long-lived nuclear waste harmful to the environment into short-lived nuclear waste, so as to reduce the storage and toxicity of radioactive waste. At present, the scientific community continues to pay attention to the research of this method. For the removal of radioactivity from nuclear power components, the removal of oxides generated on the surface of nuclear power components is another idea. Typically, about 98% of radionuclides are located in oxides on metal surfaces [4], so removing the oxides generated on the surface of nuclear power components is the key to removing their radioactivity.

Several decontamination methods have been employed, but they all have limitations. Currently used methods for decontaminating nuclear components include chemical decontamination [5], ultrasonic decontamination [6], high-pressure water jet decontamination [7], dry ice decontamination [8], and melting decontamination [9], amongst others. Chemical decontamination is a well-established technique and remains the primary method for removing radioactivity from nuclear components. Additionally, it encompasses various procedures such as reagent decontamination [10] and gel decontamination [11]. However, the disadvantage of chemical decontamination is its cumbersome operation process and the need for a variety of chemicals with different proportions. Moreover, chemical decontamination produces secondary waste, which can still threaten personnel and the environment. The ultrasonic method does not require cumbersome chemical reagents, but it has a limited removal capacity for particles that are micron-sized or smaller. High-pressure water jetting is easy to use but challenging to control, and it is only suitable for certain applications, resulting in significant amounts of radioactive waste. Dry ice decontamination can minimize secondary waste, but its cleaning power is limited, and it can only remove a thin layer of contaminants. Melting decontamination has an exceptional removal capacity, but it is energy-intensive and costly. Therefore, an efficient, energy-saving, and environmentally friendly method is necessary for the removal of contaminants on the surface of nuclear power components.

Laser decontamination is a decontamination method that can solve the above problems. The general principle of this method uses the laser’s high-energy-density characteristics to carry out laser irradiation on the surface of the substrate to achieve the removal of contaminants. Laser decontamination can be used to achieve the complete removal of pollutants without the use of any chemical reagents, even in the face of micron-level and submicron-level particles, but it also has a very good cleaning effect while avoiding excessive secondary waste. Secondly, laser decontamination can be realized via remote, non-contact operation, allowing staff to avoid contact with the experimental environment. In addition, it can be combined with CNC equipment, which can be realized automatically, reducing the hazards to personnel while improving the efficiency of the work. At present, this method of cleaning surface pollutants via laser has been applied in many fields, such as in the removal of rust [12], paint [13], metal oxidation layers [14,15], and surface deposits on cultural relics [16]. In the field of nuclear power, many scholars have also explored laser decontamination. Delaporte, P [17] developed a laser decontamination process using an excimer laser, and obtained a high decontamination effect by laser decontaminating a half-pipe taken from a steam generator of a nuclear reactor. Zhou, X [18] investigated the measurement of ablation profiles, the calculation of the ablation rate, and the chemical composition of ablated areas, and the process solution was found to be satisfactory. Kumar, A [19] investigated the change in removal depth by testing different laser power levels, laser repetition frequencies, and laser scanning speeds, as well as the number of laser processes needed to optimize the laser removal depth. Carvalho, L [20] prepared a simulated oxidized sample via direct laser irradiation and oxidation in a furnace, simulated the intrusion of radioactive elements with an europium-containing solution, and then analyzed the changes in each element based on the depth of the sample before and after laser decontamination by using glow discharge mass spectrometry (GDMS) in order to evaluate the effect of decontamination; the optimal decontamination process was obtained by varying the different laser power levels and laser scanning speeds. Xie, Y P [21] combined the finite element analysis method with COMSOL to simulate the temperature field change and ablation profile of laser single-pulse and single-line scanning, which provided a reference for the laser decontamination of actual samples, and the process parameters of laser decontamination were obtained through experimental verification. During the laser cleaning experiment, Stipp, W [22] focused on studying the infiltration of pollutants during the laser cleaning process by changing the laser fluence. This is a noteworthy issue. It is necessary to study the effect of different laser conditions on the infiltration of pollutants. Costa, P [23] used an Nd: YAG laser to treat 10 different surface pollutants on materials. He studied the removal effect of different laser fluences on pollutants. When preparing the sample, he contaminated it with a solution containing Cs elements. For nuclear power components, in addition to removing surface oxides from metal substrates, there is also laser decontamination of concrete. Oh, S Y [24] used a 10 kW fiber laser to irradiate concrete and found that the particles of concrete were sprayed due to the action of the laser.

The above studies are still in the laboratory stage, despite scholars determining the laser decontamination process parameters, the decontamination principle, and the effect of laser action on the substrate after exploring several areas of research. But for practical applications, the nuclear components we face are very large. It is not enough to consider only whether decontamination is complete. The decontamination speed is also a very important index, that is, decontamination efficiency. Most articles on laser decontamination do not emphasize the decontamination efficiency, so we calculate the laser decontamination efficiency according to the experimental parameters found in the articles on laser decontamination. Many articles also lack the relevant parameters to calculate the decontamination efficiency. From the calculation results, the laser decontamination efficiency mentioned in articles are 0.177 m^2^/h [20], 1.35 m^2^/h [25] and 4.79 m^2^/h [26], respectively. Such decontamination efficiency still cannot meet the decontamination of practical application scenarios. However, Anthofer, A [27] made a breakthrough in the efficiency of laser removal of nuclear power plant concrete. He used a 10 kW diode laser in continuous wave mode to perform laser ablation on epoxy-coated concrete, achieving a decontamination efficiency of 6.4 m^2^/h. Therefore, decontamination efficiency is particularly important for laser decontamination, which is a key to bringing laser decontamination from laboratory to practice. There is a strong need to develop a laser decontamination process that can achieve a high cleaning efficiency. In this paper, we will carry out a study where, firstly, we will prepare non-radioactive specimens by simulating the environment of the primary circuit using Alloy 690, which is a representative material for nuclear power generation, and characterize the oxidized layer of the specimens, and then we will explore the laser decontamination process with the aim of finding a laser decontamination method with a high decontamination efficiency that can be used in actual applications.

## 2. Materials and Methods

### 2.1. Materials

In this experiment, non-radioactive simulated specimens were prepared for laser decontamination by simulating the hydrochemical conditions of the nuclear power primary circuit, and the experimental material was chosen to be Alloy 690 (ASTM B166-2008 [28]), which is the main material of steam generator pipelines in nuclear power primary circuits [29]. The specific chemical composition of the material is shown in Table 1. The specimens were cut to be 30 mm × 20 mm × 2 mm rectangular flat sheets. They were sandpapered up to 1500# successively, and then were ultrasonically cleaned with anhydrous ethanol, dried, and put into a 3 L 316 stainless steel autoclave.

The corrosion specimens were prepared to accurately replicate the hydrochemical conditions of the primary circuit in a nuclear power facility. The following were the specific corrosion conditions: the concentration of boron mass was 1200 mg/L, the concentration of lithium mass was 2.2 mg/L, the temperature was kept constant at 300 °C, the pressure was 15.6 MPa, and oxidation occurred for a duration of 960 h in the autoclave.

### 2.2. Equipment

**Figure 1:**
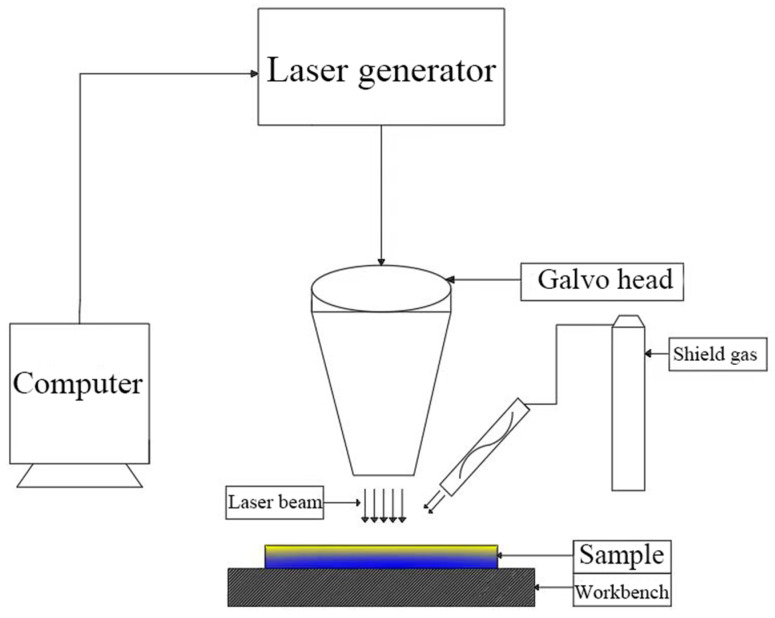
Schematic diagram of laser decontamination equipment.

Fiber lasers have become the basis of laser decontamination technology due to their high coupling efficiency, high conversion efficiency, good beam quality, etc. Therefore, the experimental equipment used for laser decontamination in this study mainly consisted of a 200 W pulsed fiber laser, a galvo head, a computer with control software, and the supporting electrical system, as shown in Figure 1. The fiber laser (YFPN-200-GMC, GZTECH, Wuhan, China) emits a circular beam with a wavelength of 1060–1080 nm and a maximum average power output of 200 W. The laser outputs Gaussian pulse laser. The galvo head is used to focus the laser beam onto the sample surface. The Alloy 690 sample is positioned on a stage capable of 100 mm movement in the X and Y directions. The computer controls the movement of the stage, allowing for the modification of laser parameters including power, scanning speed, repetition frequency, pulse width, and line spacing. Moreover, the laser pattern and scanning method (unidirectional, bidirectional, and circular filling) can be adjusted alongside the laser’s movement. In order to inhibit the secondary oxidation brought about by the transient high temperature generated by the action of the laser and sample, the experimental equipment is also equipped with nozzles, which are then connected to the gas cylinder through the gas pipe and through the flowmeter to control the speed of protective gas delivery and to provide the experimental environment with argon to isolate the oxygen. At the same time, it can also blow away the oxide particles stripped by the laser. The parameters of the fiber laser are shown in Table 2.

### 2.3. Methods

#### 2.3.1. Single-Pulse Experimental Method

**Figure 2:**
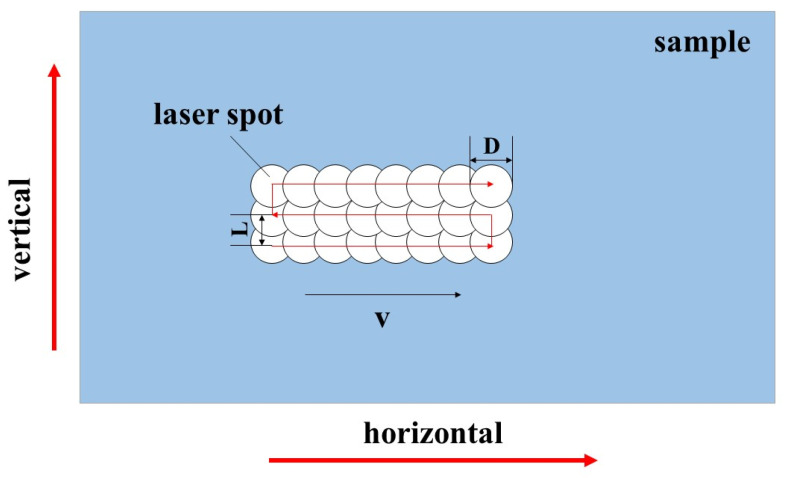
Schematic diagram of the laser decontamination process.

Laser decontamination efficiency is a measure of the speed of laser decontamination. In this paper, we use the area decontamination efficiency, which is given in the following formula [30]:

(1) $$R_{S}=\frac{S}{t}=(1-U_{p})(1-U_{L})D^{2}f,$$

where $R_{S}$ is the area decontamination efficiency, *S* is the area of laser decontamination, *t* is the laser action time, $U_{p}$ and $U_{L}$ are the transverse lap rate and longitudinal lap rate of laser decontamination, respectively, *D* is the laser spot diameter, and *f* is the laser repetition frequency. The transverse lap rate and longitudinal lap rate are expressed as follows:

(2) $$U_{P}=\left(1-\frac{v}{Df}\right)\times 100\%,$$

(3) $$U_{L}=\left(1-\frac{L}{D}\right)\times 100\%,$$

where *v* is the laser scanning speed and *L* is the line spacing in the laser scanning. The schematic diagram of the laser decontamination process is shown in Figure 2.

Therefore, the laser scanning speed and the laser line spacing become the key factors that affect the decontamination efficiency. In order to obtain a high decontamination efficiency, it is necessary to have a high scanning speed and large line spacing, which will increase the distance between the laser spot action points, even if there is no spot overlap rate. However, if it can be ensured that the effect zones of the laser can overlap with each other to completely cover the sample surface so that the removal of oxides can also be realized, then this will be the key to the success of this experiment.

In view of the above situation, the laser fluence must be determined before carrying out the laser decontamination experiments to explore the role of a single laser pulse on the sample oxide layer. In order to ensure that the single-laser-pulse range is large enough, its laser fluence must also be large enough; for Gaussian laser, its peak laser fluence formula is as follows:

(4) $$F=\frac{2P}{f\pi r^{2}},$$

where *F* is the laser fluence, *P* is the laser power, and *r* is the laser spot radius. The spot radius is fixed and the power adjustment range is also limited in order to make the laser frequency small enough. Depending on the laser operating range, the pulse width is set to 500 ns with a frequency of 40 kHz in order to realize the effect of a single pulse. The laser scanning speed is set to be fast enough, and the distance between the laser scanning single lines is far enough. By measuring the shape of the laser spot on a single line, the effect area of the laser single pulse can be measured. The scanning speed is set at 15,000 mm/s, the line spacing is 1 mm, and the laser power is used as a variable to carry out a one-factor experiment; the specific process parameters are shown in Table 3.

#### 2.3.2. Experimental Method of Laser Decontamination

It was confirmed via single-pulse experiments that adjusting the laser power, scanning speed, and line spacing can achieve complete coverage of the sample. Therefore, a laser decontamination process without spot overlapping is proposed, which will greatly improve the decontamination efficiency. According to Equations (1)–(3), if the process scheme is designed with a 40% overlap rate of the light spot, the corresponding scanning speed is 9000 mm/s and the line spacing is 0.03 mm. At this time, the decontamination efficiency is only 0.972 m^2^/h. The efficiency is very low and cannot meet the requirements in practical applications. Therefore, the scanning speed of 15,000 mm/s in the single pulse experiment is used as a parameter, and the line spacing is adjusted to 0.2 mm to ensure that the light spot can overlap. At this time, the decontamination efficiency is calculated as 10.8 m^2^/h, achieving at least a tenfold increase in efficiency. The laser decontamination power of the samples was subjected to a one-factor experiment, and the other conditions were the same as those in the process parameters of Table 3.

### 2.4. Characterization Methods

For the characterization of non-radioactive specimen oxides and the effect of laser decontamination, the macroscopic morphology of the samples was observed with a macroscopic body-viewing microscope, the microscopic morphology was observed with a field-emission scanning electron microscope (Apreo 2C, Thermo Fisher, Waltham, MA, USA), the content of each element on the surface was determined with a scanning electron microscope-supported energy spectrometer (Ultim Max, Oxford, UK), and the phase compositions of the sample surfaces before and after laser decontamination were compared using an X-ray diffractometer (Smartlab9, Rigaku (Co.) Ltd., Tokyo, Japan).

## 3. Results

### 3.1. Characterization of Non-Radioactive Specimen Oxides

**Figure 3:**
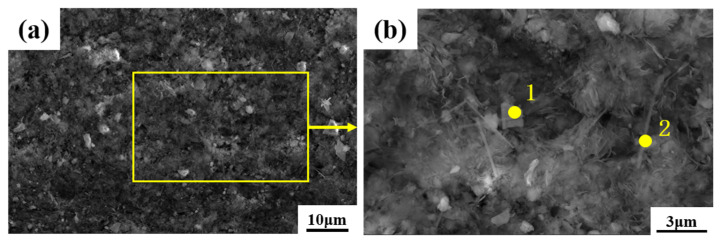
Microscopic morphology of Alloy 690 non-radioactive specimen oxides: (**a**) SEM image at 5000×; (**b**) SEM image at 20,000×.

Figure 3 shows the oxide micromorphology of the specimen observed under SEM, while Table 4 shows the comparison of the mass percentage of each element for the original specimen and the non-radioactive specimen, which serves as the basis for the evaluation of the laser decontamination results afterwards. As can be seen from Figure 3a, oxide particles are distributed on the surface of the sample, and the magnification in Figure 3b reveals that the oxides grown by Alloy 690 under primary-circuit hydrochemical conditions are polyhedral and acicular oxides, which are dispersed on the surface of the sample. The typical polyhedral and acicular oxides among them were analyzed using EDS, and the results of the atomic percentage of each element are shown in Table 5; all of them also have Ni, Cr, and Fe [31], and the polyhedral oxides have more chromium content, while the acicular oxides are predominantly nickel.

**Figure 4:**
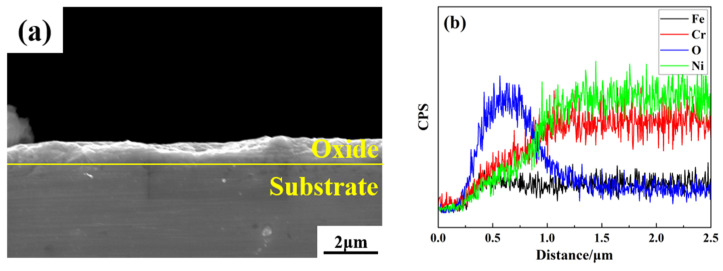
Cross-sectional morphology and elemental distribution of non-radioactive specimens of Alloy 690 oxides: (**a**) cross-section of a non-radioactive specimen; (**b**) distribution of elements in the longitudinal direction of the specimen.

The cross-sectional morphology of the oxide layer is shown in Figure 4a; the oxide layer is observed to be brighter than the substrate via SEM, and the thickness of the oxide layer is about 1 μm. Taking the longitudinal elemental distribution map in Figure 4b as an example to analyze, the thickness of the oxide layer is judged to be about 1.2 μm by combining the distribution of the elements in the five positions through the rise and fall of the elemental oxygen.

**Figure 5:**
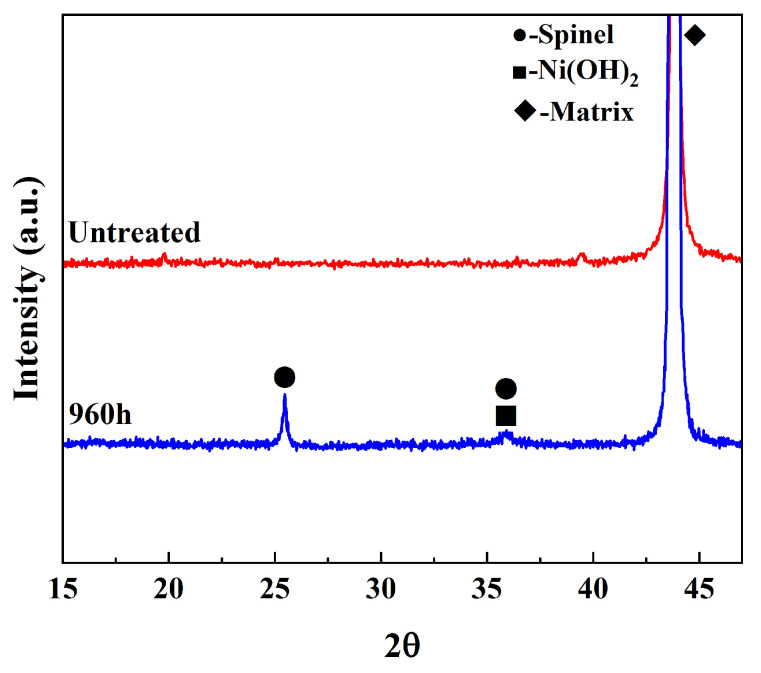
XRD comparison of non-radioactive and original specimens of Alloy 690.

Combined with the XRD analysis in Figure 5, the characteristic peaks of spinel [Ni(Fe, Cr)_2_O_4_] and Ni(OH)_2_ were observed in the spectra, and due to the thin oxidized layer of the samples, the XRD analysis also detected the characteristic peaks of the substrate, which is in agreement with the results of previous studies [32,33].

After combining the above experimental results, they can be briefly summarized as follows: the shape of the oxides produced by Alloy 690 under one-circuit hydrochemical conditions is mostly polyhedral and needle-like, and the oxide composition consists of nickel, chromium, and iron. Combined with the XRD analysis, we determined that the oxides are composed of two phases of spinel and Ni(OH)_2_; after 960 h of oxidation, the surface of the sample grows an oxide layer of about 1.2 μm.

### 3.2. Results of Single-Pulse Experiments

**Figure 6:**
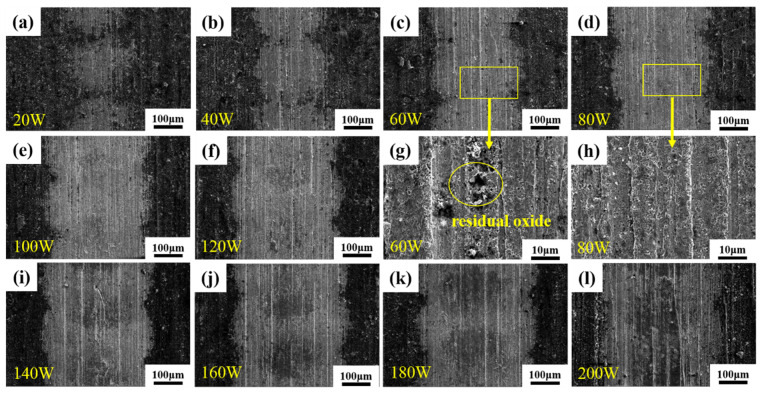
Surface morphology of laser single pulse under different levels of power: (**a**–**f**) laser power of 20–120 W; (**g**,**h**) partial enlarged diagram of the action area of laser power of 60 W and 80 W; (**i**–**l**) laser power of 140–200 W.

**Figure 7:**
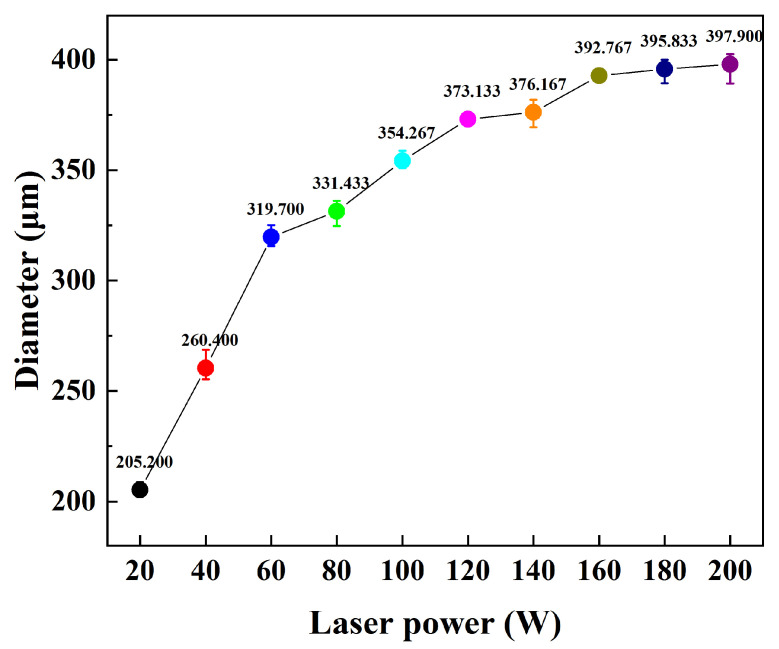
Diameter variation in single-pulse laser action area with different laser power levels.

The surface morphology of a laser single pulse with different levels of power was observed via SEM. As shown in Figure 6, the surface of the sample left traces of a circular light spot, and its action area increased with the increase in laser power. When the laser power was 20 W, the action areas did not overlap with each other, but reluctantly overlapped when it was 40 W, and then the lap range gradually increased. The diameter of the action area under each power in the figure was measured, and the data in Figure 7 were obtained. The average diameter of the action area varies from 205.2 μm to 397.9 μm. Based on Figure 6g, it can be seen that when the laser power is 60 W or less, such energy is not enough to completely remove the oxides on the sample surface, and residual oxides are also observed on the surface; however, after 80 W, no residual oxides can be seen on the sample surface (Figure 6h), so the removal threshold of oxides under this condition can be determined to be 80 W (203.72 J/cm^2^).

**Figure 8:**
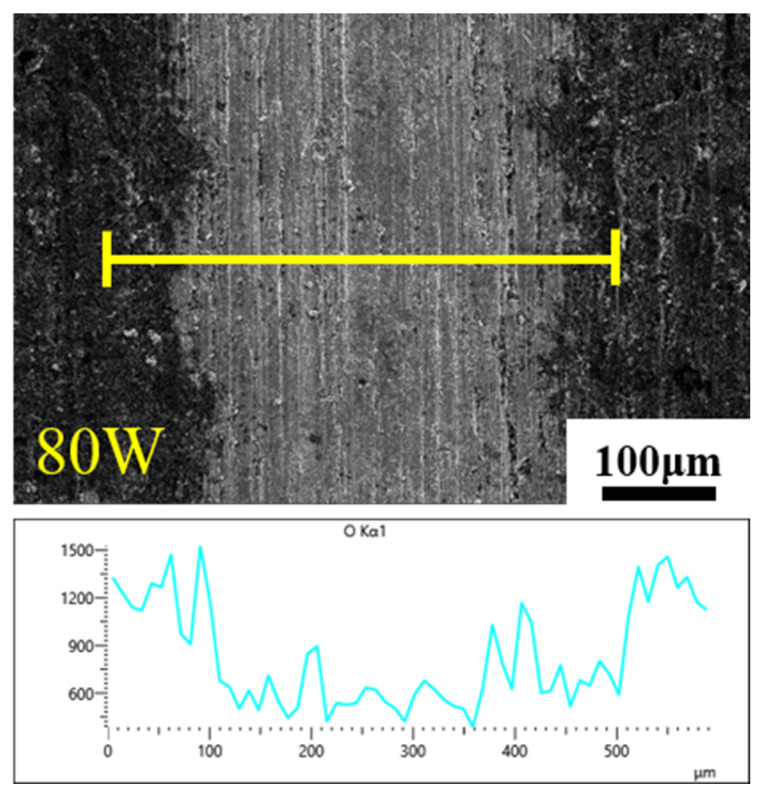
Distribution of oxygen between the zone of action and the oxide layer at a laser power of 80 W.

Taking the laser power of 80 W as an example, line scanning under EDS was carried out in the laser action area and the surrounding oxide layer area, and the changes in the oxygen element in these two areas were analyzed. As shown in Figure 8, the distribution of the oxygen element shows a trend of first decreasing and then increasing. Compared with the oxide layer, the oxygen element content in the laser action area has been greatly reduced, and the average content is only 7%, which achieves the effect of removing oxides.

Combining the above findings, it can be concluded that increasing the laser power leads to a rise in laser fluence. Moreover, when the laser fluence reaches a specific value, it enables the complete eradication of oxides, determining the oxide removal threshold. Additionally, an increase in the laser power extends the action field, making it feasible to create a process with a high decontamination efficiency.

### 3.3. Surface Morphology after Laser Decontamination

#### 3.3.1. Macroscopic Morphology

**Figure 9:**
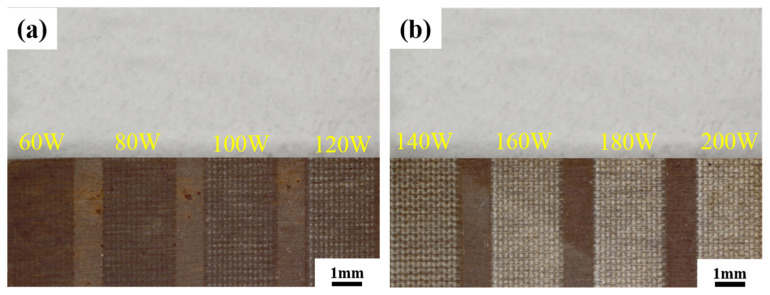
Macroscopic morphology of sample after single-factor experiment using different laser decontamination power levels. (**a**) laser power 60–120 W; (**b**) laser power 140–200 W.

Figure 9 shows the macroscopic surface morphology of the sample after the laser decontamination experiment. It can be clearly seen that the spot characteristics are arranged neatly, which is the result of the action of a single-pulse laser. There are gaps between the action areas, which are residual oxides that are not affected by the laser. With the increase in laser power, the laser action area gradually increases, the gap gradually shrinks, and the surface becomes brighter as it gradually approaches the color of the metal substrate. Under the power level of 160 W, the laser action area completely covers the gap, and the oxide layer is basically removed. Thus, 160 W is considered as the best power level for the laser removal of oxides from the non-radioactive sample of Alloy 690.

#### 3.3.2. Microscopic Morphology

**Figure 10:**
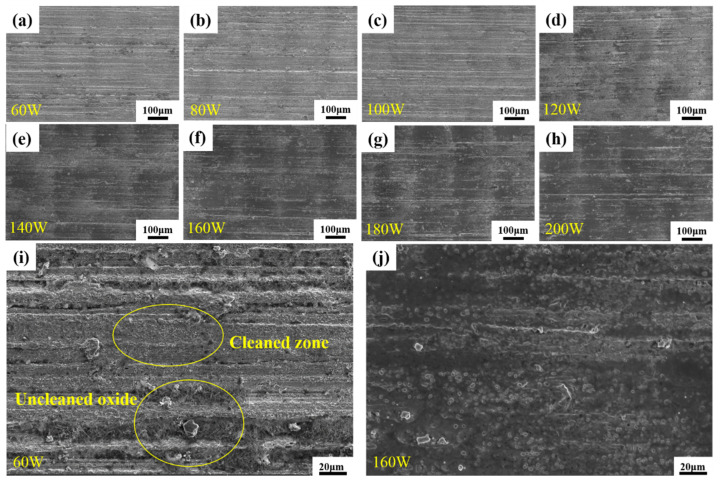
Microscopic morphology of samples after single-factor experiment using different laser power levels: (**a**–**h**) photos at 500× with a laser power of 60–200 W; (**i**,**j**) photos at 2500× with a laser power of 60 W and 160 W.

Figure 10 shows the microscopic morphology of the sample observed with a scanning electron microscope after the laser decontamination experiment, and obvious traces after the laser action can be seen. With the increase in laser power, the range of the laser action becomes larger, and the surface changes from gray to black, which represents the gradual decrease in oxides. When the samples treated with a laser power of 60 W and 160 W are magnified to 2500×, it can be clearly seen in Figure 10i (60 W) that the oxides are removed in the area of the action center of the laser spot, but there are oxides around it that are not affected by the laser, which proves that the removal is incomplete. In Figure 10j (160 W), it can be observed that no residual oxide particles can be seen on the surface of the sample, but some holes appear on the surface of the sample, which is presumed to be related to the laser action. The existence of these holes is also why a gray color can still be seen on the surface although the laser power reaches 160 W. Thus, the purpose of removing the oxides has been achieved at this time.

### 3.4. Elemental Analysis

**Figure 11:**
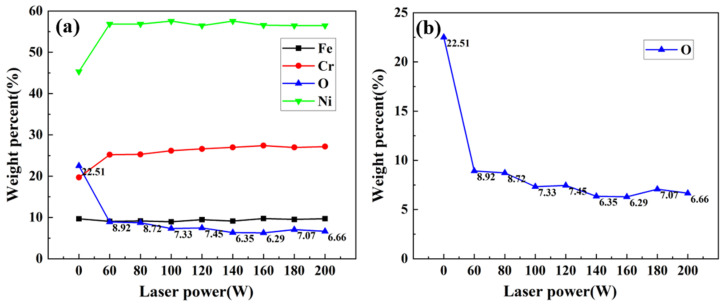
Average contents of various elements on the surface of samples under different laser power levels: (**a**) changes in the content of major elements; (**b**) changes in the content of oxygen.

Figure 11 shows the average content of each element on the sample surface under different laser power levels. For nickel-based alloys mainly containing nickel, chromium, and iron, the change in iron content is not obvious, while the change in nickel, chromium, and oxygen is the opposite.

Focusing on the change in oxygen content (Figure 11b), the oxygen content drops from 22.51% at the beginning to below 9% after laser irradiation, which shows that the laser can remove oxides at this time, but there are residual oxides on the surface due to the limited laser action area, so the oxygen content is high.

With the further increase in laser power, the area of the laser action is enlarged, causing the oxide residue to decrease. The oxygen content is kept below 7% from 140 W until it drops to 6.29% when the laser power is 160 W, which is the lowest of all power levels. Then, the slight increase in oxygen content may be caused by the secondary oxidation phenomenon caused by the high temperature of the laser. Based on the above experimental results, the optimum laser power is determined to be 160 W.

**Figure 12:**
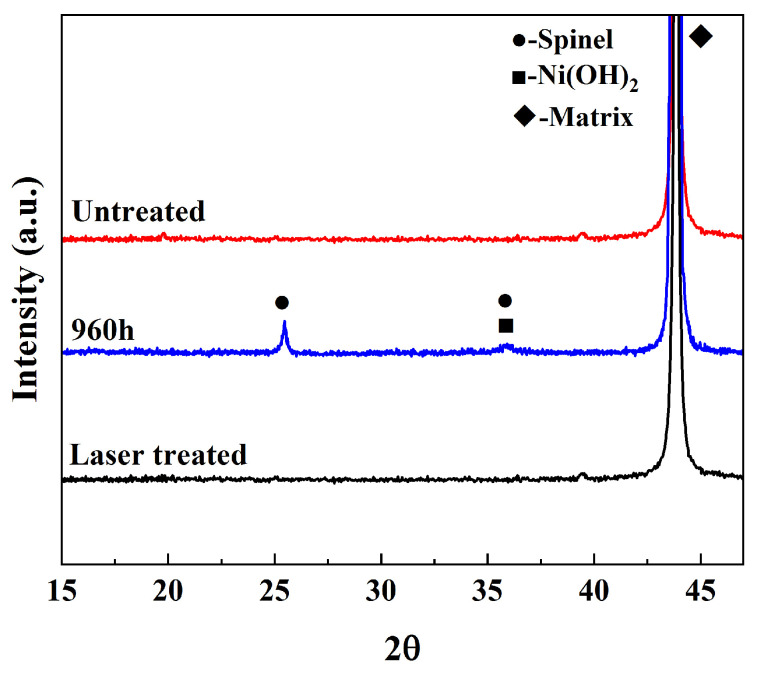
XRD comparison before and after laser decontamination at 160 W power.

Then, the sample under the optimum power condition was analyzed via XRD, and the results are shown in Figure 12. Compared with the oxidized sample, the characteristic peak of the oxides has disappeared, and the XRD spectrum of the sample after laser decontamination is consistent with the original sample, which proves that the laser has removed the oxides on the surface of the sample.

## 4. Discussion

In this experiment, a process with a high decontamination efficiency of 10.8 m^2^/h was realized by exploring laser decontamination of the oxide layer on the surface of non-radioactive specimens of Alloy 690. According to the results of the decontamination (Figure 6 and Figure 7), a single pulse of the laser can produce a circular area with a diameter of nearly 400 μm and a laser spot diameter of only 50 μm, which leads to a completely new idea: as long as the overlap condition of the laser action area is reached, the removal of the oxide layer on the surface of the sample can be realized without overlapping the laser spot, which will greatly enhance the efficiency of the laser decontamination process. As described in the design of the experimental scheme, the decontamination efficiency is only 0.972 m^2^/h under the condition of 40% light spot overlapping rate designed by the decontamination system. The decontamination efficiency of the non-spot overlapping process reaches 10.8 m^2^/h, achieving at least 10 times improvement. In the face of large nuclear power components later, we can use a smaller power laser to achieve higher efficiency of laser decontamination. Such process design can save energy consumption and cost.

In addition, a 200 W pulsed fiber laser was used for this experiment. In the field of laser cleaning, the application of pulsed lasers accounts for the vast majority [17,18,19,20,21,22,23]. Song, K H [34] used a single-mode continuous fiber laser to remove contaminants from the surface of stainless steel and Anthofer, A [27] used a 10 kW diode laser in continuous wave (CW) mode to perform laser ablation on epoxy-coated concrete. Compared to continuous-wave lasers, pulsed lasers can achieve higher energy density, resulting in better cleaning effectiveness. Additionally, the pulsing mode does not lead to large heat accumulation on the substrate surface, thereby minimizing damage to the material. Furthermore, due to its high coupling efficiency and good beam quality, fiber laser has been widely used in the field of laser cleaning, compared to CO_2_ lasers [35] and excimer lasers [17].

The principle of laser decontamination will be further discussed in this section. According to previous studies, the main principle of laser decontamination is the thermal expansion of the substrate for the removal of oxide particles from the surface of a sample [36,37]. For nanoseconds (ns) and longer pulses (μs or ms), the absorption of laser energy by the substrate is mainly driven by a thermal diffusion mechanism [38]. When the pulsed laser interacts with the substrate, it causes the temperature to increase, causing the thermal expansion of the substrate, which leads to mechanical stresses and inertial forces. For oxide particles, the three main adhesion forces are van der Waals, capillary, and electrostatic forces, where the van der Waals forces prevail at the micrometer and sub-micrometer scales [39]. If this effect of thermal expansion of the substrate exceeds the adhesion force between the particles and the substrate, then the particles will detach from the substrate [40]. If the pulse energy is high enough, the surface contaminants can also be removed via evaporation [38], which explains the ablative effect of the laser.

Returning to the present experiment, the oxides within the irradiated area of the laser spot are removed through melting or evaporation due to the absorption of the laser energy, which causes the rise in temperature that reaches the melting or boiling point, while for the oxides outside the irradiated area, the removal principle is due to the thermal expansion of the substrate.

The irradiation of the laser causes a force to be generated by heating an area larger than the irradiation area. When this force is greater than that of the adhesion of the oxide layer, the stripping of the oxide layer is realized; thus, this explains the phenomenon where the laser’s area of action is larger than the spot area.

## 5. Conclusions

In the present work, the process of laser decontamination of the oxide layer on the surface of non-radioactive specimens prepared under simulated one-loop aqueous chemical conditions was investigated using a pulsed fiber laser. A process with a high decontamination efficiency was explored, and the results of the decontamination were evaluated. In addition, the effect of different laser power levels on the surface morphology of the material as well as on the elemental composition was studied. The main conclusions can be listed as follows:

1. Alloy 690 specimens with a boron mass concentration of 1200 mg/L and a lithium mass concentration of 2.2 mg/L, under conditions of a temperature of 300 °C, a pressure of 15.6 MPa, and continuous oxidation for 960 h in a autoclave, produced an oxide layer consisting of polyhedral and acicular particles of nickel–chromium–iron oxides with a thickness of about 1.2 μm, which consisted of two composition phases: spinel and Ni(OH)_2_.
2. The laser pulse width was set as 500 ns, the frequency was 40 kHz, the scanning speed was 15,000 mm/s, and the line spacing was 1 mm for the laser single-pulse experiments. The laser power levels ranged from 20 W to 200 W, corresponding to the laser fluence of 50.93 J/cm^2^ to 509.3 J/cm^2^, and changes in the average diameter of the single-laser-pulse area ranged from 205.2 μm to 397.9 μm, which is larger than the diameter of the laser spot. The laser fluence of 203.72 J/cm^2^, corresponding to a laser power of 80 W, was determined as the oxide removal threshold.
3. Keeping the above parameters unchanged, the line spacing was changed to 0.2 mm for the laser decontamination experiments, at which time the decontamination efficiency was 10.8 m^2^/h. When the laser power was changed from 60 W to 200 W, the surface of the samples became brighter and its color was close to that of the substrate, while the microscopic oxide particles were gradually reduced and the content of the elemental oxygen was gradually reduced from the original 22.51% to 6.29%.
4. By comparing the effects of different power levels after decontamination, it was determined that the laser power level of 160 W was the best power level. At this time, the oxides on the surface of the sample were removed, and the oxygen content was 6.29%. The XRD spectrum after decontamination was consistent with that of the original substrate, with a good decontamination effect. It is proved that the high decontamination efficiency of 10.8 m^2^/h can remove the surface oxides from a non-radioactive Alloy 690 specimen oxidized for 960 h.

## Figures and Tables

**Table 1:** Chemical composition of Alloy 690 (wt%).

Element	Ni	Cr	Fe	Cu	Mn	Si	C	S	P
wt%	62.9	28.2	8.4	0.20	0.17	0.12	0.02	0.005	0.003

**Table 2:** Parameters of the fiber laser.

Technical Parameter	Unit	Indicators
Product type		YFPN-200-GMC
Maximum power	W	200
Power adjustment range	%	10–100
Pulse width	ns	10–500
Center wavelength	nm	1060–1080
Focusing spot diameter	μm	50
Cooling method		Air cooling
Working temperature	°C	0–40

**Table 3:** Process parameters of the laser single-pulse experiment ^1^.

Power (W)	Laser Fluence/(J·cm^−2^)
20	50.93
40	101.86
60	152.79
80	203.72
100	254.65
120	305.58
140	356.51
160	407.44
180	458.37
200	509.30

^1^ The other process parameters for the single pulse experiment are: pulse width of 500 ns, frequency of 40 kHz, speed of 15,000 mm/s, line spacing of 1 mm, focusing spot diameter of 50 μm.

**Table 4:** Composition comparison of Alloy 690 base material and surface oxides.

Elemental Mass Percentage %	O	Fe	Cr	Ni
Alloy 690	0	8.40	28.20	63.30
960 h	22.51	9.68	19.69	45.32

**Table 5:** Atomic percentage of Alloy 690 oxide elements.

Elemental Atomic Percentage %	O	Fe	Cr	Ni
Polyhedral 1	60.19	4.92	13.45	21.44
Acicular 2	55.35	6.25	8.64	29.76

## Data Availability

The data presented in this study are available on request from the corresponding author. The data are not publicly available due to privacy reasons.
